# Recognition

**Published:** 1917-12

**Authors:** E. C. Kirk


### "RECOGNITION"

BY E. C. KIRK, D.D.S.

In our previous issue we discussed the principle involved in the exemption of dental students from active service, and incidentally an opinion attributed to the Hon. Secretary of War, in effect that "If necessary, the army could get along without dentists"—a point of view manifestly due to lack of knowledge of the function and purpose of dental service as related to the health and efficiency of the army. That the opinion thus expressed does not reflect the views of our national legislative bodies, the medical profession nor of the general public is practically demonstrated by the fact that when the statement quoted was made, there was pending in the Senate a bill specifically recognizing in principle the importance of dental service to the health of the army personnel on exactly the same plane as the national government has accorded to every other department of the medical service. During the closing hours of the 65th Congress this bill, the text of which appears in this issue (see page 1169), became law by executive approval. Briefly stated, the law provides that within the Dental Surgeons Corps there shall be "commissioned officers of the same grade and proportionately distributed among such grades as are now or may be hereafter provided by law for the Medical Corps, who shall have the rank, pay, promotion, and allowances of officers of corresponding grades in the Medical Corps, including the right to retirement as in the case of other officers." The same law further provides for the relief of drafted dental students from active military duty during the time necessary to the completion of their college professional courses on the same basis as students in medicine have been likewise permitted to complete their professional training.

Taken together, these two enactments of our national government mark an epoch in the history of dentistry. From the time of its organization as a professional activity in 1839—indeed, even before that period—those whose tastes and inclination led them to specialize in the study of the mouth and teeth had demanded recognition for their field of activity as a legitimate department of medical science and art. This demand has been persistent and continuous, and has been with equal persistence rejected and combated by the organized medical profession because from the medical point of view the dentist, by reason of his special training and his special degree, lacked the hallmark of legitimacy as the practitioner of a recognized medical specialty. Medicine assumed with questionable justification that any devotee of the science and art of healing who had not pursued the accepted and conventional curriculum of training that terminated in the medical degree was *ipso facto* unqualified to practice any specialty of the healing art, nor was his judgment even with regard to his special field entitled to respectful attention by those who held the medical qualification.

The demand for recognition of the dental specialty is based upon its intrinsic importance as a department of the healing art. The fathers—the pioneers who laid the foundations of the plan which gave to dentistry its professional status—with clear insight and prophetic vision and with unswerving faith in the soundness and justice of their position, were content to work out their professional salvation in the belief that time and the inherent merit of their cause would eventually bring to it the recognition to which it was in equity entitled. Progress toward the goal has been tardy, and often needlessly impeded by the human factors of personal ambition and prejudice, nevertheless the advance, though slow, has been sure, and the end has been inevitable from the first.

The growth of scientific knowledge by which the interrelationships of the mouth structures and teeth and the general bodily economy have been made manifest by the researches of dental and medical investigators, has served to close up and in large measure to obliterate the hiatus between dentistry and medicine. It has been discovered that dentistry comprises more than dental art and craftmanship, just as it was discovered long ago that there is more in surgery than mere handicraft; that medical science is the basal foundation of dentistry just as it is the groundwork of surgery and the unifying factor of all of the special departments of healing. The contributions of dentistry to the science of medicine in the general sense of that term have made it an admitted department of medicine. The artificial and conventional distinction which has heretofore caused it to be regarded as a "thing apart" has gradually undergone disintegration until it has become a distinction in name only and no longer a distinction in fact.

That professional prejudice, the instinct of self-preservation, fanatical adherence to an ancient and hopelessly senile ideal, on the part of the medical profession, has largely delayed, though it could not prevent, the ultimate recognition of dentistry as a legitimate medical specialty is now a demonstrated fact. But the altered state of mind of society which is being wrought out by the democratizing effect of the world war has suddenly wrought the change which in normal times would have waited a generation longer for fulfillment. The service of dentistry in the conservation of health has become recognized as one of the indispensable factors in the winning of the war; and in order that dental service may become effective it has naturally followed that it must be relieved of the handicap which subserviency to superior rank in medical administration has heretofore imposed upon it in its army relationships. The Congress of the United States has therefore in its wisdom cut the Gordian knot of professional prejudice and opened the way for efficient dental service for those engaged in the defensive work of the nation. The official act of the national Congress is therefore the mark of an epoch in dentistry—the official baptism of the dental specialty as a recognized department of the science and art of healing.

The achievement of our goal is not to be regarded as one of the accidents of political activity. Its attainment was only possible because dentistry by scientific investigation had made known the causal relationships of dental and oral pathological conditions to disorders of the general health—that bodily health is in its degree dependent upon oral hygiene as it is upon sound health in other parts of the body. The demonstration has been made and official recognition accorded.

We have at other times cited the principle that every opportunity carries with it a corresponding responsibility. The application of that principle to the present case is manifest. If we are to maintain and permanently justify the recognition which has been accorded us, our achievement in scientific professional progress for the future must keep pace with—indeed, it must exceed—what it has been in the past. ''We have our 'blue china;' now we must live up to it.'' We are facing ''the next step,'' and the answer to ''Will dentistry take it?'' is yet to come. —Editorial in November *Cosmos.*

